# Proteinase 3-antineutrophil cytoplasmic antibody-positive necrotizing crescentic glomerulonephritis complicated by infectious endocarditis: a case report

**DOI:** 10.1186/s13256-019-2287-1

**Published:** 2019-12-05

**Authors:** Katsunori Yanai, Yoshio Kaku, Keiji Hirai, Shohei Kaneko, Saori Minato, Yuko Mutsuyoshi, Hiroki Ishii, Taisuke Kitano, Mitsutoshi Shindo, Haruhisa Miyazawa, Kiyonori Ito, Yuichiro Ueda, Masahiro Hiruta, Susumu Ookawara, Yoshihiko Ueda, Yoshiyuki Morishita

**Affiliations:** 1 Division of Nephrology, First Department of Integrated Medicine, Saitama Medical Center, Jichi Medical University, 1-847 Amanuma-cho, Omiya-ku, Saitama, Saitama 330-8503 Japan; 2 Division of Pathology, First Department of Integrated Medicine, Saitama Medical Center, Jichi Medical University, Saitama, Japan; 3 Department of Diagnostic Pathology, Dokkyo University Koshigaya Medical Center, Saitama, Japan

**Keywords:** Necrotizing crescentic glomerulonephritis, Infective endocarditis, Proteinase 3-antineutrophil cytoplasmic antibody

## Abstract

**Background:**

Proteinase 3-antineutrophil cytoplasmic antibody has been reported to be positive in 5–10% of cases of renal injury complicated by infective endocarditis; however, histological findings have rarely been reported for these cases.

**Case presentation:**

A 71-year-old Japanese man with a history of aortic valve replacement developed rapidly progressive renal dysfunction with gross hematuria and proteinuria. Blood analysis showed a high proteinase 3-antineutrophil cytoplasmic antibody (163 IU/ml) titer. *Streptococcus* species was detected from two separate blood culture bottles. Transesophageal echocardiography detected mitral valve vegetation. Histological evaluation of renal biopsy specimens showed necrosis and cellular crescents in glomeruli without immune complex deposition. The patient met the modified Duke criteria for definitive infective endocarditis. On the basis of these findings, the patient was diagnosed with proteinase 3-antineutrophil cytoplasmic antibody-positive necrotizing crescentic glomerulonephritis complicated by *Streptococcus* infective endocarditis. His renal disease improved, and his proteinase 3-antineutrophil cytoplasmic antibody titer normalized with antibiotic monotherapy.

**Conclusion:**

Few case reports have described histological findings of proteinase 3-antineutrophil cytoplasmic antibody-positive renal injury complicated with infective endocarditis. We believe that an accumulation of histological findings and treatments is mandatory for establishment of optimal management for proteinase 3-antineutrophil cytoplasmic antibody-positive renal injury complicated with infective endocarditis.

## Background

Proteinase 3-antineutrophil cytoplasmic antibody (PR3-ANCA) has been reported to be positive in 5–10% of cases of renal injury complicated by infective endocarditis [1]; however, histological findings have rarely been reported for these cases. In addition, the clinical course and optimal treatment have not been fully clarified.

We report a case of a patient with rapidly progressive PR3-ANCA-positive necrotizing crescentic glomerulonephritis complicated by *Streptococcus* infective endocarditis. The patient’s renal disease improved with antibiotic therapy without any immunosuppressive agents, and his PR3-ANCA titer normalized in accordance with improving infective endocarditis.

## Case presentation

Our patient was a 71-year-old Japanese man who had undergone the Bentall procedure and biological aortic valve replacement for the treatment of descending aortic aneurysm and aortic regurgitation at 70 years of age. Thereafter, his renal function had been normal (serum creatinine level, 0.93 mg/dl) without hematuria and proteinuria. Two months before admission, he had appetite loss, malaise, and gross hematuria. One month before admission, he noticed purpura on his lower extremities. A laboratory examination conducted by his primary care physician showed anemia (hemoglobin, 9.2 g/dl), thrombocytopenia (platelet count, 10 × 10^4^/μl), hematuria, and proteinuria. Therefore, he was referred to our hospital for further management.

**Fig. 1:**
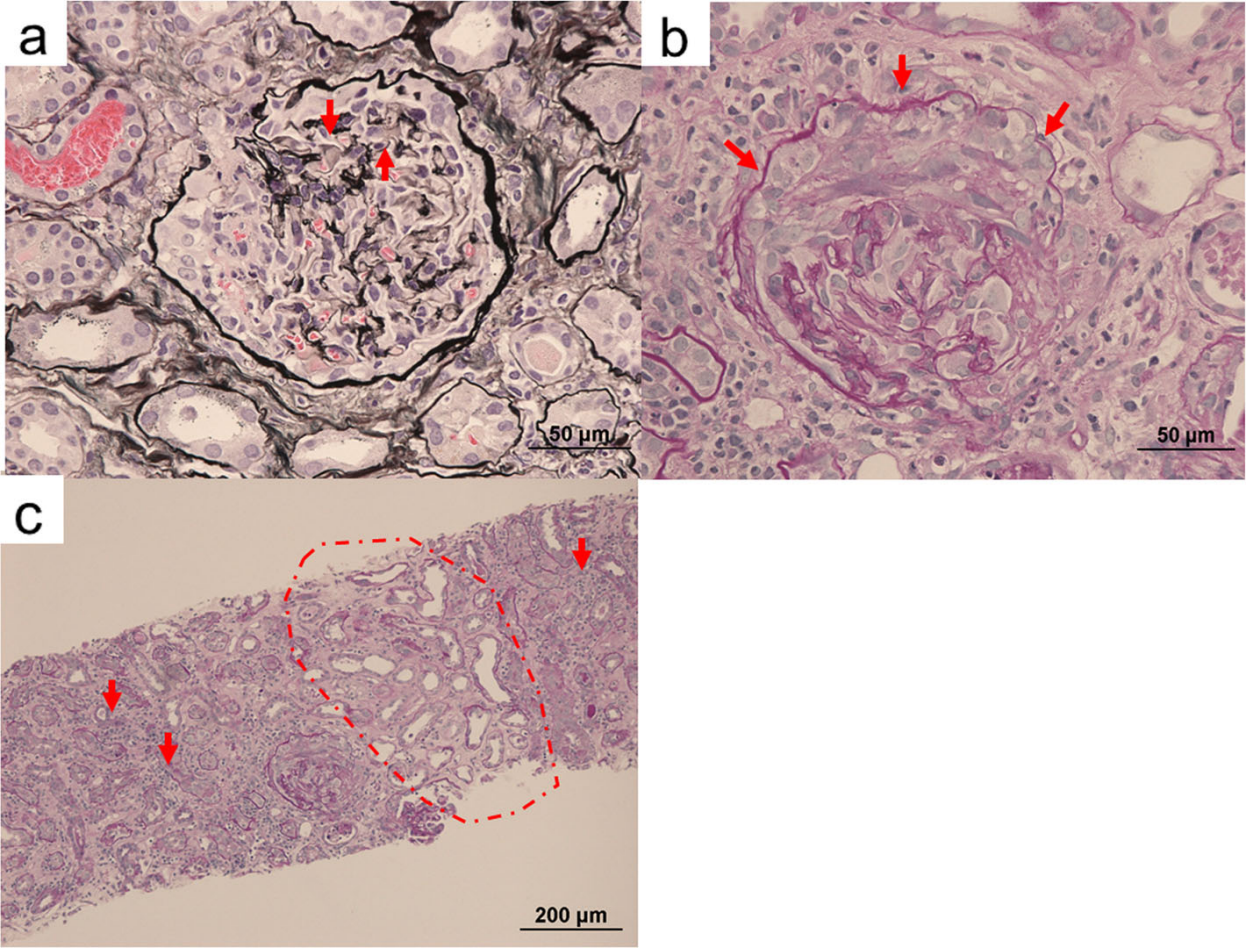
Renal biopsy findings. **a** Glomerulus with partial fibrinoid necrosis with fragmentation of glomerular tufts (*arrows*) (periodic acid-methenamine silver stain; original magnification, 400×). **b** Glomerulus with cellular crescentic formation (*arrows*) (periodic acid-Schiff stain; magnification, original magnification, 400×). **c** Tubulointerstitium with sporadic neutrophil infiltration in the peritubular capillary (*arrows*) and atrophy (*broken line*) (periodic acid-Schiff stain; original magnification, 100×)

**Fig. 2:**
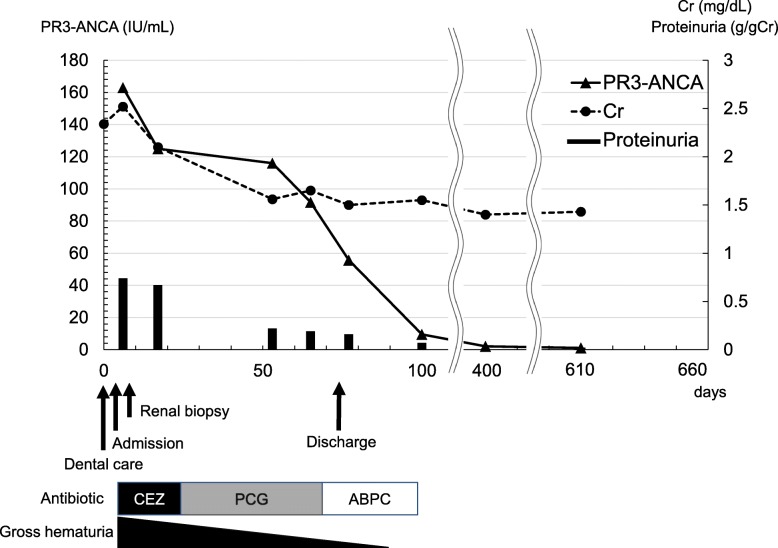
The patient’s clinical course. *ABPC* Ampicillin, *CEZ* Cefazolin, *Cr* Creatinine, *PCG* Penicillin G, *PR3-ANCA* Proteinase 3-antineutrophil cytoplasmic antibody

Upon admission, his body temperature was 36.9 °C, and his blood pressure was 120/60 mmHg. Anemia, edema, and symmetrically distributed palpable purpura of the lower extremities were observed. He had no characteristic physical findings of infective endocarditis, such as Osler nodes, Roth spots, and Janeway lesions. Cardiac auscultation revealed 2/6 systolic reflux murmur at the cardiac apex. Blood analysis showed that the patient’s serum creatinine level was elevated at 2.34 mg/dl, and his serum hemoglobin level was reduced at 7.6 g/dl. Urinalysis showed proteinuria at 0.74 g/g Cr and microscopic hematuria. PR3-ANCA level was elevated at 163 IU/ml (normal range, < 10 IU/ml). The patient had negative test results for hepatitis B antigen, hepatitis C antibody, cryoglobulin, antistreptolysin O, antinuclear antibody, immune complex, and myeloperoxidase-ANCA. Serum complement C3 was mildly decreased, whereas C4 was normal. Laboratory data obtained at admission are summarized in Table 1. No abnormalities were found in the patient’s chest x-ray or electrocardiogram. *Streptococcus* species was detected from two separate blood culture bottles. On the third hospital day, renal biopsy was performed. Histological analysis revealed that 54% (6 of 11) of glomeruli showed partial fibrinoid necrosis with fragmentation of glomerular tufts (Fig. 1a), and 27% (3 of 11) of glomeruli showed cellular crescents (Fig. 1b). No fibrocellular or fibrous crescents and no endocapillary proliferation were found. The mesangium showed no increase in cells or matrix. The tubulointerstitium partially showed neutrophilic and lymphocytic infiltration in the peritubular capillary and atrophy (Fig. 1c). Fibrinoid necrosis was not observed in vessel walls. Immunofluorescence microscopy showed no deposition of immunoglobulins and complement factors. Electron microscopy showed small amounts of nonspecific electron-dense deposits in subendothelial areas and the paramesangial area. At this point, the patient met the modified Duke criteria for definitive infective endocarditis [2] (mitral valve vegetation on echocardiography, two positive blood cultures of *Streptococcus* species drawn 3 days apart, glomerulonephritis). On the eighth hospital day, transesophageal echocardiography revealed mitral valve vegetation. On the 12th hospital day, spinal magnetic resonance imaging showed pyogenic spondylitis at T7/T8 and L4/L5. On the basis of these findings, the patient was diagnosed with rapidly progressive PR3-ANCA-positive necrotizing crescentic glomerulonephritis complicated by *Streptococcus* infective endocarditis. Antibiotic therapy including cefazolin and penicillin G followed by oral administration of ampicillin was provided without immunosuppressive agents. Thereafter, his renal disease, endocarditis, and pyogenic spondylitis improved. He was discharged from our center on the 73rd hospital day. He has since received regular outpatient treatment in our department. At 7 months after discharge, his serum creatinine level had decreased to 1.43 mg/dl, his proteinuria had decreased to 0.15 g/g Cr, and his hematuria had decreased to 1.1 red blood cells per high-power field. His PR3-ANCA level had decreased to within the normal range (Fig. 2).

**Table 1:** Laboratory findings upon admission

Complete blood count and blood chemistry	Value
WBC	13,600/μL
Bands	2%
Segments	82%
Eosinophils	0%
Basophils	0%
Lymphocytes	7%
Monocytes	8%
RBC	323 × 10^4^/μL
Hemoglobin	7.6 g/dL
Hematocrit	29.2%
Platelet	12.0 × 10^4^/μL
Total protein	7.0 g/dL
Albumin	2.6 g/dL
AST	35 IU/L
ALT	23 IU/L
CRP	7.57 mg/dL
Na^+^	130 mmol/L
K^+^	5.3 mmol/L
Cl^−^	101 mmol/L
Ca^2+^	7.7 mg/dL
Phosphate	3.8 mg/dL
BUN	25 mg/dL
Cr	2.52 mg/dL
eGFR	20.8 ml/minute/1.73 m^2^
Uric acid	6.3 mg/dL
HbA1c	6.3%
Glucose	106 mg/dL
ASO	73 IU/mL
Hepatitis B antigen	< 0.04
Hepatitis C antibody	< 0.29
IgG	2692 mg/dL
IgA	340 mg/dL
IgM	350 mg/dL
Anti-DNA antibody	< 10
Anti-RNP antibody	Negative
Anti-Sm antibody	Negative
C3	50 mg/dL
C4	17 mg/dL
CH50	23.4 U/mL
Antinuclear antibody	160
PR3-ANCA	163 IU/mL
MPO-ANCA	< 1.0 IU/mL
Anti-GBM antibody	< 2.0 IU/mL
ESR	86 mm/hour
Rheumatoid factor	86 IU/mL
Urinary analysis	
RBC	Numerous (dysmorphic)/HPF
WBC	1–4/HPF
Protein	0.74 g/g Cr
β_2_-MG	12,133 μg/L

*Abbreviations*: *ALT* alanine aminotransferase, *ASO* antistreptolysin O, *AST* aspartate aminotransferase, *β*_*2*_*-MG* β_2_-microglobulin, *BUN* blood urea nitrogen, *CH50* 50% homolytic unit of complement, *Cr* creatinine, *CRP* C-reactive protein, *C3* complement component 3, *C4* complement component 4, *eGFR* estimated glomerular filtration rate, *ESR* erythrocyte sedimentation rate, *GBM* antiglomerular basement membrane antibody, *HbA1c* hemoglobin A1c, *HPF* high-power field, *Ig* immunoglobulin, *MPO-ANCA* myeloperoxidase antineutrophil cytoplasmic antibody, *PR3-ANCA* proteinase 3 antineutrophil cytoplasmic antibody, *RBC* red blood cells, *RNP* ribonucleoprotein, *Sm* Smith, *WBC* white blood cells

## Discussion and conclusions

We report a case of rapidly progressive PR3-ANCA-positive necrotizing crescentic glomerulonephritis complicated by *Streptococcus* infective endocarditis. The patient’s renal disease improved with antibiotic monotherapy, which led to normalization of PR3-ANCA titer in accordance with improving infective endocarditis.

Renal disease associated with infective endocarditis shows various pathological changes including crescent formation, fibrinoid necrosis, mesangial cell proliferation, and endothelial cell thickening in the glomerulus and tubulointerstitial damage with infiltration of immune cells [3–7]. PR3-ANCA has been reported to be positive in 5–10% of cases of renal disease complicated with infective endocarditis [1]. It is considered that PR3-ANCA may be produced as a result of an immune response against infection by sharing epitopes with cytoplasmic antigens of neutrophils in cases of infective endocarditis [8]. The produced PR3-ANCA is then speculated to contribute to fibrinoid necrosis, crescent formation, and granulomas in the kidney [9]. However, the lack of sufficient histological findings of PR3-ANCA-positive renal diseases complicated by infective endocarditis prevents clarification of detailed pathological changes in the kidney. Although many cases of PR3-ANCA-positive renal disease complicated by infective endocarditis have been reported, including crescentic glomerulonephritis, endocapillary proliferative glomerulonephritis, mesangial proliferative glomerulonephritis, and focal segmental glomerulonephritis, only three cases showed necrotizing crescentic glomerulonephritis complicated by infective endocarditis [10–34] (Table 2). Regarding treatment for PR3-ANCA-positive renal disease complicated by infective endocarditis, previous studies suggested antibiotic monotherapy for patients with low PR3-ANCA titers (< 25 IU/ml) and combination therapy with immunosuppressive agents, including steroids for patients with high PR3-ANCA titers (> 50 IU/ml), when the patients’ condition does not improve with antibiotic monotherapy [22, 35]. The three previous cases of PR3-ANCA-positive necrotizing crescentic glomerulonephritis showed various PR3-ANCA titers (2.96, > 8.0, and 85 IU/ml) and were treated with immunosuppressive agents such as corticosteroids in addition to antibiotics. Among those three cases, the renal disease resolved completely in two patients but progressed to end-stage renal disease in the other (Table 2). The other types of PR3-ANCA-positive renal disease complicated by infective endocarditis also showed various PR3-ANCA titers (3–359 IU/ml) and were treated with antibiotics with or without immunosuppressive agents (Table 2). Regarding treatment outcomes, most renal diseases recovered, except for one patient with crescentic glomerulonephritis with high PR3-ANCA titers (247 IU/ml) and one patient with mesangial proliferative glomerulonephritis with high PR3-ANCA titers (143 IU/ml), both of whom died (Table 2). In our patient, necrotizing crescentic glomerulonephritis improved with antibiotic monotherapy, and PR3-ANCA titer normalized in accordance with improving infective endocarditis; however, PR3-ANCA titer was highly elevated at 163 IU/ml. The results of our patient’s case suggest that antibiotic monotherapy can be effective even if the PR3-ANCA titer is considerably high in PR3-ANCA-positive necrotizing crescentic glomerulonephritis complicated by infective endocarditis. However, caution is needed with the use of immunosuppressive agents because they may exacerbate bacteremia and infective endocarditis. Furthermore, a greater accumulation of cases with histological evidence is needed to investigate optimal treatments for PR3-ANCA-positive renal disease complicated with infective endocarditis.

**Table 2:** Case reports of PR3-ANCA-positive renal injury complicated by infective endocarditis

Age (years)/sex	Renal biopsy histology (IF/EM)	PR3-ANCA (IU/mL)	Microbe detected	Past medical history	Treatments	Outcome	Reference
54/M	Focal necrotizing crescentic GN (negative/no deposits)	2.96	*Streptococcus mutans*	MVP	Piperacillin, tazobactam, cyclophosphamide, corticosteroids	Complete recovery	[10]
59/M	Focal necrotizing crescentic GN (negative/no deposits)	> 8.0	*Enterococcus faecalis*	Hepatitis B and C	Pulse methylprednisolone ⇒ prednisolone	Hemodialysis	[11]
67/M	Focal necrotizing crescentic GN (IgA^+^, IgM^+^, IgG^+^, C3^+^, C1q^+^/mesangial and subendothelial dense deposits)	85	*Gemella sanguinis*	No mention	Ceftriaxone, gentamicin, methylprednisolone	Temporary hemodialysis ⇒ complete recovery	[12]
6/M	Crescentic GN (no mention)	Positive	*Bartonella henselae*	CHD	Doxycycline, rifampicin	No mention	[13]
12/F	Crescentic GN (C3^+^/subendothelial dense deposits)	Positive	*Gemella morbillorum*	Nothing	Penicillin, gentamicin, steroids	Complete recovery	[14]
14/M	Crescentic GN (no mention)	Positive	*Bartonella henselae*	CHD	Doxycycline	No mention	[13]
18/F	Crescentic GN (IgG[1+], IgM[3+], C3[2+], C1q[2+]/subendothelial dense deposits)	32	*Bartonella henselae*	Tetralogy of Fallot, SSS, and complete heart block	Antibiotic therapy, methylprednisolone	Complete recovery	[15]
24/M	Crescentic GN (C3^+^/mesangial and subendothelial dense deposits)	14	α-Hemolytic *Streptococcus*	VSD	Cefotaxime, prednisolone	No renal dysfunction	[16]
26/F	Crescentic GN (IgM^+^ C3^+^/no mention)	160	*Streptococcus viridans*	ASD, recent dental Tx	Penicillin G, tobramycin	Recovery	[17]
36/M	Crescentic GN (negative/no mention)	359	*Bartonella henselae*	No mention	Prednisolone, intravenous cyclophosphamide ⇒ azathioprine, MMF, prednisolone, CV surgery	Complete recovery	[18]
43/M	Crescentic GN (negative/no mention)	Positive	*Bartonella henselae*	Infective endocarditis (blood culture was negative)	Cyclophosphamide, prednisoloneCV surgery	No mention	[19]
46/M	Crescentic GN (C3^+^ C1q^+^/no mention)	25	Negative	No mention	Amoxicillin, gentamicin, penicillin	Complete recovery	[20]
47/M	Crescentic GN (IgM^+^, IgA^+^, C3^+^, C1q^+^/mesangial and subendothelial dense deposits)	160	*Bartonella henselae*	Cat scratch disease	Doxycycline, rifampicin 6 weeks	Recovery	[21]
50/M	Crescentic GN (negative/not performed)	247	*Streptococcus oralis*	Nothing	Steroid therapy ⇒ ampicillin, gentamicin, vancomycin	Death	[22]
54/M	Crescentic GN (IgM^+^, C3^+^/no deposits)	3	*Streptococcus mutans*	No mention	Ampicillin ⇒ vancomycin, corticosteroids, cyclophosphamide	Recovery	[23]
55/M	Crescentic GN (C3[2+], IgA^+^/no deposits)	> 8.0	*Bartonella henselae, Bartonella quintana*	Depression	Vancomycin, cefepime ⇒ doxycycline, rifampicin, methylprednisolone	Recovery	[24]
67/M	Crescentic GN (IgM^+^, C3^+^, C1q^+^/no mention)	41	*Bartonella henselae*	Thoracic aortic aneurysm repair	Rifampicin, doxycycline, methylprednisolone	Temporary hemodialysis ⇒ recovery	[25]
72/F	Crescentic GN (no mention)	Positive	*Aggregatibacter aphrophilus*	No mention	Vancomycin, ceftriaxone	Temporary hemodialysis ⇒ recovery	[26]
42/M	Diffuse endocapillary proliferative GN (C3^+^/subendothelial dense deposits)	21.3	*Staphylococcus aureus*	Nothing	Cefazolin	Recovery	[27]
68/M	Diffuse endocapillary proliferative GN and crescentic GN (IgG[2+], IgM[3+], C3[3+], C1q[2+]/subendothelial dense deposits)	102	Negative	Schistosomiasis	Cefoperazone, tazobactam	Complete recovery	[28]
78/F	Endocapillary proliferative GN (IgM^+^, C3^+^, C1q^+^/no mention)	30	*Bartonella henselae*	Hypertension	Doxycycline	Complete recovery	[29]
48/M	Mesangial proliferative GN (C3^+^/no mention)	12	Negative	Alcoholism, DM	Amoxicillin, gentamicin	Complete recovery	[20]
57/M	Mesangial proliferative GN (IgG^+^, IgM^+^, C3^+^ /no mention)	45	Negative	Nothing	Corticosteroids ⇒ ampicillin, ceftriaxone, gentamicin, vancomycin	Recovery	[30]
74/M	Mesangial proliferative GN (IgG^+^, C1q^+^/not performed)	> 100	*Bartonella henselae, Bartonella quintana*	IHD, pacemaker, DM, pulmonary embolus	Antibiotic therapy	Recovery	[31]
78/M	Mesangial proliferative GN (IgM^+^, C3^+^, IgA^+^, C1q^+^/subendothelial dense deposits)	143	*Enterococcus faecalis*	Coronary artery bypass surgery	Antibiotic therapy	Death	[32]
57/M	FSGS (IgM^+^, C3^+^/paramesangial dense deposits)	40	Negative	DM, AVR, aortic aneurysm	Pulse methylprednisolone ⇒ vancomycin, gentamicin, rifampicin	Plasmapheresis ⇒ recovery	[33]
64/M	FSGS and mild interstitial inflammation (no mention)	60	Negative	Insidious mild renal dysfunction	Ceftriaxone, doxycycline	Complete recovery	[34]

*Abbreviations*: *ASD* atrial septal defect, *AVR* aortic valve replacement, *CHD* chronic heart disease, *C3* complement component 3, *CV* cardiovascular, *DM* diabetes mellitus, *EM* electron microscopy, *F* female, *FSGS* focal segmental glomerulonephritis, *GN* glomerular nephritis, *IF* immunofluorescence, *IHD* ischemic heart disease, *Ig* immunoglobulin, *LM* light microscopy, *M* male, *MMF* mycophenolate mofetil, *MVP* mitral valve prolapse, *PR3-ANCA* proteinase 3 antineutrophil cytoplasmic antibody, *SSS* sick sinus syndrome, *Tx* treatment, *VSD* ventricular septal defect

In conclusion, we describe a case of a patient with PR3-ANCA-positive necrotizing crescentic glomerulonephritis complicated by infective endocarditis. His renal disease was improved with antibiotic agents, and his PR3-ANCA titer normalized in accordance with improving infective endocarditis.

## Abbreviations

ABPC: Ampicillin
ALT: Alanine aminotransferase
ASD: Atrial septal defect
ASO: Antistreptolysin O
AST: Aspartate aminotransferase
AVR: Aortic valve replacement
β_2_-MG: β_2_-Microglobulin
BUN: Blood urea nitrogen
C3: Complement component 3
C4: Complement component 4
CEZ: Cefazolin
CH50: 50% Homolytic unit of complement
CHD: Chronic heart disease
Cr: Creatinine
CRP: C-reactive protein
CV: Cardiovascular
DM: Diabetes mellitus
eGFR: Estimated glomerular filtration rate
EM: Electron microscopy
ESR: Erythrocyte sedimentation rate
F: Female
FSGS: Focal segmental glomerulonephritis
GBM: Antiglomerular basement membrane antibody
GN: Glomerular nephritis
HbA1c: Hemoglobin A1c
HPF: High-power field
IF: Immunofluorescence
Ig: Immunoglobulin
IHD: Ischemic heart disease
LM: Light microscopy
M: Male
MMF: Mycophenolate mofetil
MPO-ANCA: Myeloperoxidase antineutrophil cytoplasmic antibody
MVP: Mitral valve prolapse
PCG: Penicillin G
PR3-ANCA: Proteinase 3-antineutrophil cytoplasmic antibody
RBC: Red blood cells
RNP: Ribonucleoprotein
Sm: Smith
SSS: Sick sinus syndrome
Tx: Treatment
VSD: Ventricular septal defect
WBC: White blood cells
